# Alpha-actnin-4 (ACTN4) selectively affects the DNA double-strand breaks repair in non-small lung carcinoma cells

**DOI:** 10.1186/s13062-022-00354-6

**Published:** 2022-12-07

**Authors:** Daria Kriger, Ksenia Novitskaya, Giomar Vasileva, Ekaterina Lomert, Nikolai D. Aksenov, Nikolai A. Barlev, Dmitri Tentler

**Affiliations:** 1 Institute of Cytology, Russian Academy of Sciences, Tikhoretsky Ave. 4, St Petersburg, Russian Federation 194064; 2 Nazarbayev University, 020000 Astana, Kazakhstan

**Keywords:** ACTN4, Non-small cell lung cancer (NSCLC), Etoposide resistance, DNA repair, Non-homologous end joining (NHEJ), Homologous recombination (HR)

## Abstract

**Background:**

ACTN4 is an actin-binding protein involved in many cellular processes, including cancer development. High ACTN4 expression is often associated with a poor prognosis. However, it has been identified as a positive marker for platinum-based adjuvant chemotherapy for non-small cell lung cancer (NSCLC). The goal of our study was to investigate the involvement of ACTN4 in the NSCLC cells’ response to the genotoxic drugs.

**Results:**

We generated H1299 cells with the ACTN4 gene knock-out (ACTN4 KO), using the CRISPR/Cas9 system. The resistance of the cells to the cisplatin and etoposide was analyzed with the MTT assay. We were also able to estimate the efficiency of DNA repair through the DNA comet assay and gamma-H2AX staining. Possible ACTN4 effects on the non-homologous end joining (NHEJ) and homologous recombination (HR) were investigated using pathway-specific reporter plasmids and through the immunostaining of the key proteins. We found that the H1299 cells with the ACTN4 gene knock-out did not show cisplatin-resistance, but did display a higher resistance to the topoisomerase II inhibitors etoposide and doxorubicin, suggesting that ACTN4 might be somehow involved in the repair of DNA strand breaks. Indeed, the H1299 ACTN4 KO cells repaired etoposide- and doxorubicin-induced DNA breaks more effectively than the control cells. Moreover, the ACTN4 gene knock-out enhanced NHEJ and suppressed HR efficiency. Supporting the data, the depletion of ACTN4 resulted in the faster assembly of the 53BP1 foci with a lower number of the phospho-BRCA1 foci after the etoposide treatment.

**Conclusions:**

Thus, we are the first to demonstrate that ACTN4 may influence the resistance of cancer cells to the topoisomerase II inhibitors, and affect the efficiency of the DNA double strand breaks repair. We hypothesize that ACTN4 interferes with the assembly of the NHEJ and HR complexes, and hence regulates balance between these DNA repair pathways.

**Supplementary Information:**

The online version contains supplementary material available at 10.1186/s13062-022-00354-6.

## Background

The alpha-actinin 4 (ACTN4) was originally described as an actin-binding cytoskeletal protein associated with cancer cell motility [1]. Since then, ACTN4 was found to be involved in a variety of cellular processes, including cell mobility [2], regulation of the cell cycle [3] and growth [4], epithelial-to-mesenchymal transition [5], retrovirus replication [6], activation of transcription factors [7, 8] and of nuclear receptors [9, 10]. Despite of this multifunctionality, mutations in the ACTN4 gene lead to a specific type of hereditary renal dysfunction, FSGS, although underlying mechanisms of this disease are still under investigation [11–13].

Mechanistically, ACTN4 interacts with several dozen proteins, both in the nucleus and in the cytoplasm. Besides actin, the list of interactants includes NF-kappaB, beta-catenin, estrogen receptor, hnRNP family members, chromatin remodeling proteins INO80 and HDAC7 [3, 5, 7, 14–18]. These proteins are responsible for regulation of multiple processes, often in a cell-type specific manner. Despite the functional meanings of these interactions are not always fully understood, it is thought that they may mediate pleiotropic, but tissue-specific, effects of ACTN4.

There are a number of studies investigating the effects of ACTN4 on cancer development. Clinical data show that overexpression often correlates with a poor prognosis, metastasis, and an invasive phenotype (see reviews [19, 20]). Amplification of the ACTN4 gene located on the 19q chromosome has also been detected in patients with pancreas [21], ovary [17], lung [22], and salivary gland [23] tumors. Nevertheless, a low level of ACTN4 is observed in the prostate tumors, while high ACTN4 expression inhibits the proliferation of the cancer cells [14]. High ACTN4 expression has been identified as a marker of the platinum-based therapy outcome in lung adenocarcinoma patients. A significant clinical benefit to overall patient survival, when using the cisplatin-based adjuvant chemotherapy, was detected in the patient group with the high ACTN4 expression level [24, 25]. Curiously, no change in cisplatin resistance has been observed in the A549 non-small cell lung cancer (NSCLC) line after the ACTN4 gene knock-down [24].

In the present study, we further investigated possible ACTN4 involvement in the NSCLC cells’ resistance to genotoxic drugs. Our results showed that the H1299 cells with ACTN4 depletion were not cisplatin-resistant, but did display higher resistance to the topoisomerase II inhibitors. We demonstrate that ACTN4 may be involved in the DNA strand breaks repair. Moreover, the data suggests that ACTN4 may interfere with the assembly of the non-homologous end joining (NHEJ) and homologous recombination (HR) protein complexes, and hence regulates the balance between these double strand break repair pathways.

## Results

### The ACTN4 knock-out increases resistance of the H1299 cells to genotoxic stress

**Fig. 1:**
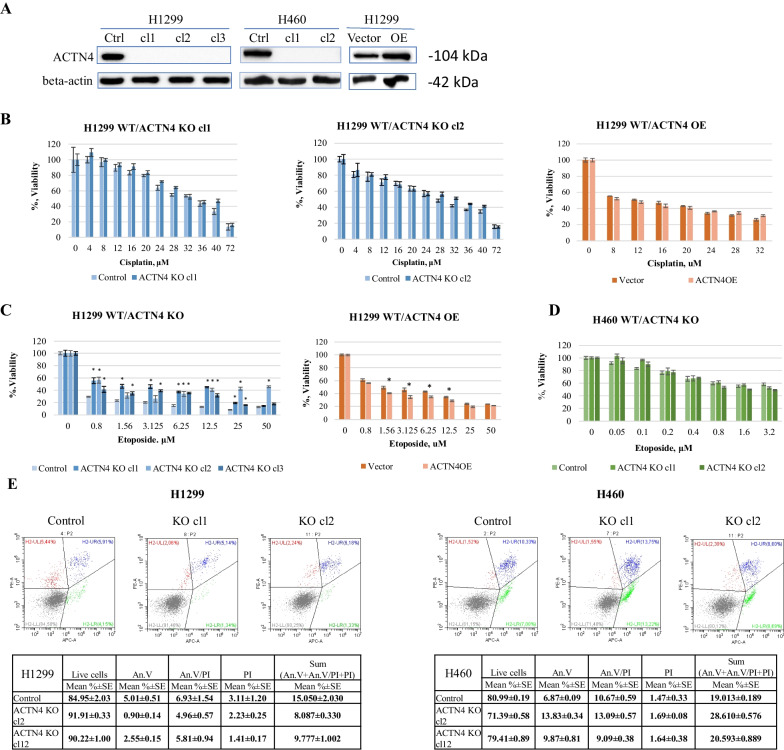
The ACTN4 knock-out affects the viability of H1299 cells under genotoxic stress. **A** Western Blot analysis of ACTN4 expression levels in H1299 (left) and H460 (right) control and ACTN4 knock-out (ACTN4 KO) cells. Three H1299 and two H460 ACTN4 KO clones used in the study are presented. **B**–**D** The MTT assay performed on H1299 (**B**, **C**) and H460 (**D**) control, ACTN4 KO and ACTN4 OE cells exposed by different concentrations of cisplatin (**B**) and etoposide (**C**, **D**) for 72 h. Results for three H1299 ACTN4 KO clones are presented in **C** to ensure the effect. **E** The H1299 (left) and H460 (right) cells were treated by 50 uM etoposide for 24 h to induce apoptosis. Annexin V (APC)/PI (PE) plots and percentages of cells in quadrants (means of three replicas) are presented. H1299 ACTN4 KO cells display significantly (*p* < 0.001) lower early apoptosis (AV) than the control cells while H460 cells show the opposite. The data are presented as the mean of at least three replicas ± SEM. ***p < 0.001, **p < 0.01, *p < 0.05 compared to untreated cells (multiple Student’s t-test). Each assay was performed at least three times

According to recent studies, overexpression of the ACTN4 gene may be a positive marker for the application of platinum-based therapy in lung adenocarcinoma patients [24, 25]. We decided to test the involvement of ACTN4 in the response of lung cancer cells to DNA damaging drugs with different mechanisms of action. To do that, we generated NSCLC cells H1299 with knock-out of the ACTN4 gene (ACTN4 KO) using the CRISPR/Cas9 system (see “Materials and Methods” Section). As a result, several clones were obtained that showed significant attenuation of the ACTN4 protein level (Fig. 1A). Next, we estimated the effect of ACTN4 knock-out on the sensitivity of H1299 cells to cisplatin and to etoposide. The former one is the platinum-based agent that introduces various crosslinks within DNA molecules, whereas the latter is a topoisomerase II inhibitor, mainly inducing double-strand breaks (DSB). Both agents have been used for the treatment of patients with NSCLC [26]. Sensitivity of the control H1299 cells and ACTN4 KO clones to cisplatin (4–72 uM) and etoposide (0.8–50 uM) was compared by MTT assay. The cells were treated for 3 days. Surprisingly, we did not observe a correlation between ACTN4 expression and cell sensitivity to cisplatin at any concentration tested (Fig. 1B). Nevertheless, the ACTN4 knock-out clones showed significant resistance to etoposide, compared to the control cells (Fig. 1C left). Six knock-out clones were tested to ensure the effect. The first two were chosen for the follow-up experiments. Furthermore, we constructed H1299 cell line with ACTN4 overexpression (ACTN4 OE), and found that they were less resistant to etoposide then control cells (Fig. 1C right). Next, we generated ACTN4 knock-out clones in another NSCLC cell line, H460, that have more epithelial phenotype and differ from H1299 cells by their p53 and KRAS status (Fig. 1A). However, no significant difference in sensitivity to etoposide between the clones and control cells was observed. Next, we evaluated the apoptosis rate in both H1299 and H460 cell lines as a major cause of the cell death after genotoxic stress. The results confirmed the lower apoptosis rate in H1299 ACTN4 KO clones treated with etoposide (Fig. 1E, left panel), which coincided with the MTT data. Curiously, etoposide caused higher apoptosis in H460 ACTN4 KO clones compared to the control cells (Fig. 1E, right panel). Considering that no difference has been revealed with the MTT test, the results suggest that some other ACTN4 effects may cause the higher apoptosis rate in H460 cells.

**Fig. 2:**
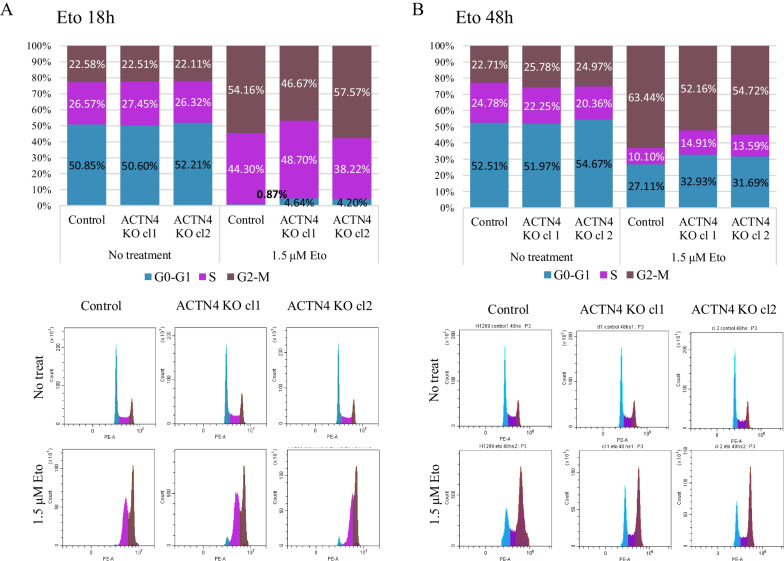
ACTN4 knock-out does not change cell cycle in H1299 cells but increases G0-G1 phase under genotoxic conditions. H1299 Control and ACTN4 KO cells were treated with 1.5 uM etoposide for 18 (**A**) or 48 (**B**) hours. Cell cycle distributions are presented as graphs (upper panels) and flow cytometry plots (lower panels). The data are presented as the mean of at least three replicas in each experiment ± SEM and repeated three times

Since a higher resistance of the ACTN4 KO cells to etoposide may result from the cell cycle changes, we compared the cell cycle distribution in the control and knock-out cells. The analysis revealed that the depletion of ACTN4 had no effect in the H1299 cells under normal conditions (Fig. 2A). However, treatment with etoposide caused G1 phase augmentation. Thus, we decided to investigate the effect of ACTN4 on DNA damage-induced cell cycles in more details. First, the H1299 control and ACTN4 KO cells were treated with 1.5 uM etoposide for 18 h to ensure that all cells passed once through the cycle. The vast majority of cells were observed in S- and G2/M-phase (Fig. 2A). The G1 phase of the cell cycle was nearly absent in the control cells, compared to 5% in H1299 ACTN4 KO clones. In the second variant, the cells were treated for 48 h, so they could overcome the effect of cell cycle arrest and return to cycle as in the MTT assay. We observed an approximately even distribution between the G1-phase and G2/M-phase, Again, about 5% more cells in the G-phase were detected in the ACTN4 KO clones in comparison to the control cells (Fig. 2B).

Thus, we found that the down-regulation of the ACTN4 gene increases the resistance of H1299 cells to genotoxic stress caused by the etoposide but not by the cisplatin. Moreover, ACTN4 overexpression showed the opposite effect on sensitivity to etoposide. Although, the ACTN4 gene knock-out does not affect the cell cycle per se, we observe a less profound cell cycle arrest after the etoposide treatment. Collectively, the data suggest that ACTN4 may somehow interfere with the cells’ responses to the etoposide-induced DNA damage.

### The ACTN4 KO cells more efficiently repair DNA breaks induced by the topoisomerase II inhibitors

**Fig. 3:**
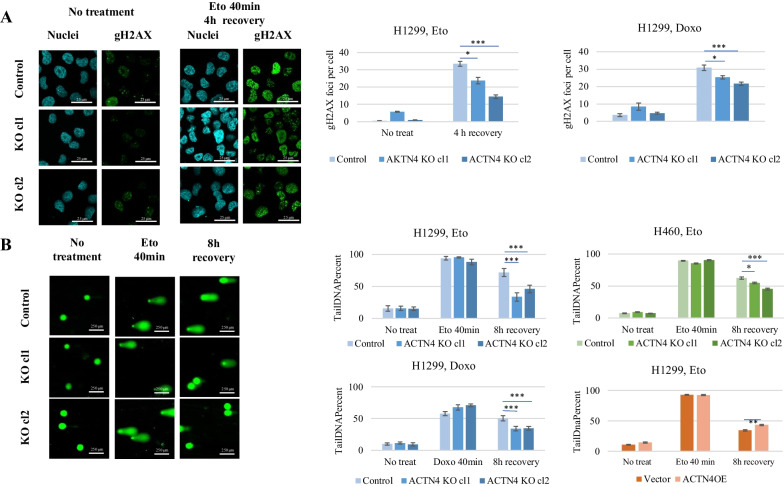
Depletion of ACTN4 in H1299 and H460 cells leads to more efficient DNA break repairs induced by the topoisomerase II inhibitors. Control, ACTN4 KO and ACTN4 OE cells were treated with 50 uM etoposide (Eto) or 1.5 uM doxorubicin (Doxo) for 40 min. The drug was then replaced with fresh media and 4 or 8 h of cells recovery. **A** Analysis of gamma-H2AX immunostaining in H1299 control and ACTN4 KO cells. Images of the etoposide-induced foci formation are presented in the left panel. Mean numbers of foci per nucleus were calculated in Eto or Doxo treated cells. The graphs represent the mean gamma-H2AX foci numbers between three replicas (± SE) with at least 500 cells per replica analyzed. Scale bars in panel represent 25 μm. **B** An alkaline comet assay is performed on H1299 and H460 cells. Images of the DNA comets obtained from H1299 control, ACTN4 KO and ACTN4 OE cells are presented in the left panel. Scale bars in panel represents 250 μm. The graphs represent mean Tail DNA percentages of at least 100 analyzed cells ± SE. ***p < 0.001, *p < 0.05 compared to untreated cells (multiple Student’s t-test). Each assay was performed at least three times. The experiments were performed in triplicate

In order to investigate possible ACTN4 involvement in the DNA repair process, we examined the formation of the gamma-H2AX histone foci as a commonly used marker of the DNA damage response [27]. To assess the specificity of cell response to etoposide, we included another topoisomerase II inhibitor, doxorubicin, in the analysis. The H1299 control and ACTN4 KO cells were treated with either etoposide (50 uM) or doxorubicin (1.5 uM) for 40 min to induce the DNA breaks. The genotoxic agents were then removed, and the cells were incubated 4 h more. The number of gamma-H2AX foci was evaluated by immunostaining followed by an analysis with a high-content image analysis system (see “Materials and Methods” Section). We found that the mean number of gamma-H2AX foci per nucleus were reduced in H1299 ACTN4 KO clones compared to the control cells (Fig. 3A). Importantly, similar results were observed using etoposide and doxorubicin. Attenuation of the gamma-H2AX signal in ACTN4 knockout cells may suggest that the efficiency of the DSBs repair is enhanced in this case.

To ensure that the observed differences in gamma-H2AX staining results from a more effective repair of the DNA lesions rather than defective recognition of the DNA breaks [28], we estimated the DNA integrity in repairing cells using DNA comet assay. The experimental design was similar to the one described above. The H1299 control cells, the ACTN4 KO clones and ACTN4 OE cells were treated with etoposide or doxorubicin for 40 min, and the repair efficiency was analyzed after 8 h. The alkaline comet assay showed that DNA was significantly damaged after the etoposide and doxorubicin treatments compared to the intact DNA of non-treated cells (Fig. 3B). At the same time, no correlation between the DNA damage and the ACTN4 gene expression was observed. After removing the genotoxic agents, the DNA lesions were progressively repaired but the ACTN4 KO clones demonstrated significantly less DNA damage than the control cells (Fig. 3B). Both etoposide and doxorubicin treatments revealed similar results. Moreover, a higher DSBs repair rate was also observed in H460 ACTN4 KO clones (Fig. 3B, upper right graph). On the contrary, H1299 ACTN4 OE cells revealed more DNA damage, which supports correlation between the ACTN4 expression level and the DSBs repair efficiency (Fig. 3B, lower right graph).

Our data indicates that ACTN4 may negatively affect the DSBs repair machinery. Hence, better resistance of H1299 ACTN4 KO cells to etoposide treatment occurs due to the higher efficiency of the DNA repair process relatively to the control cells. Similarly, higher DNA repair efficiency was detected in H460 ACTN4 KO cells.

### ACTN4 depletion determines the DSBs repair pathway, promoting non-homologous end joining and suppressing homologous recombination

**Fig. 4:**
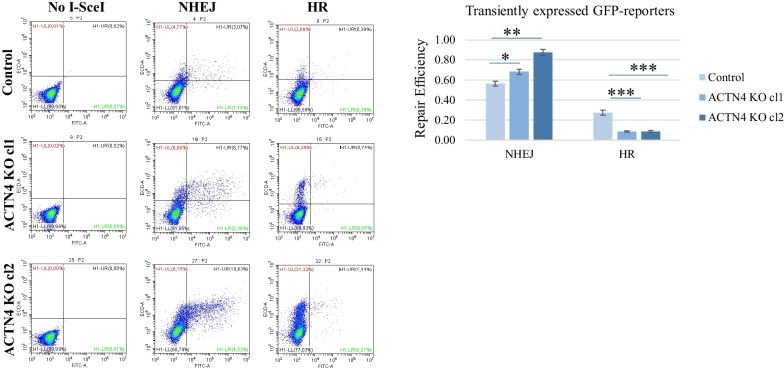
ACTN4 cells show enhanced NHEJ and suppressed HR. H1299 cells were transfected with NHEJ and HR specific reporter constructs along with a plasmid, encoding dsRed-fused I-SceI endonuclease that performs site-specific cleavage of the reporters. Representative FACS plots display NHEJ and HR specific repair of the reporter GFP as an increase of GFP-positive cells (upper quadrants). The graph represents DNA repair efficiency estimated as the ratio of GFP + /dsRed + cells. Data are presented as mean of three replicas ± SD. ***p < 0.0001compared to untreated cells (Student’s t-test). The assay was performed three times

Next, we investigated the possible effects of ACTN4 on two major DSBs repair systems, homologous recombination (HR) and non-homologous end joining (NHEJ). In order to directly evaluate the contribution of NHEJ and HR to overall DNA repair in control and ACTN4 KO cells, we used the fluorescence reporter-based approach described in [29]. Briefly, the GFP gene is inactivated by inserts that may be removed if digested with the I-SceI endonuclease and then repaired with either NHEJ or HR, respectively. We transiently transfected H1299 control and ACTN4 KO cells with the NHEJ- or HR-specific reporter plasmids and the I-SceI-GR-RFP plasmid, encoding I-SceI endonuclease fused with the ligand binding domain of the glucocorticoid receptor (GR), which, in the absence of ligands, anchored the endonuclease to the cytoplasm. To ease the visualization of the restriction enzyme, I-SceI-GR was fused to RFP fluorescent proteins. To induce the translocation of the I-SceI-GR-RFP fusion to the nucleus and the cleavage of the reporter plasmids, we added a synthetic corticosteroid triamcinolone acetonide. Successful repair of the I-SceI-induced breaks by NHEJ or HR restored the functionality of the GFP gene [29]. The number of GFP-positive cells were analyzed after three days post-transfection by flow cytometry, using a green-versus-red fluorescent plot. Consequently, the efficiency of the NHEJ and HR was estimated as percentage of GFP-positive cells normalized to the percentage of the RFP-positive cells. The results showed that ACTN4 KO clones had more efficient NHEJ but suppressed HR pathways compared to the control H1299 cells (Fig. 4). To assess the involvement of chromatin in the ACTN4-mediated DNA repair, we generated stable control and ACTN4 KO cell lines that carried NHEJ and HR reporter plasmids. The cell lines were then transfected with the I-SceI-GR-RFP plasmid, and the GFP/RFP ratio was analyzed as described above. The results showed that the percentage of the GFP-positive cells was about 4–5 times lower compared to the transient transfection. Nevertheless, the overall trend remained similar, i.e., in ACTN4 KO clones the NHEJ repair efficiency was higher, and HR was lower, than in control cells (Additional file 1: Figure S1). Thus, the data suggests that low ACTN4 expression shifts the ratio between NHEJ and HR in favor of the former pathway. Since NHEJ is a more frequent event (Fig. 4), it may explain the higher efficiency of the DSBs repair in the H1299 ACTN4 KO cells.

**Fig. 5:**
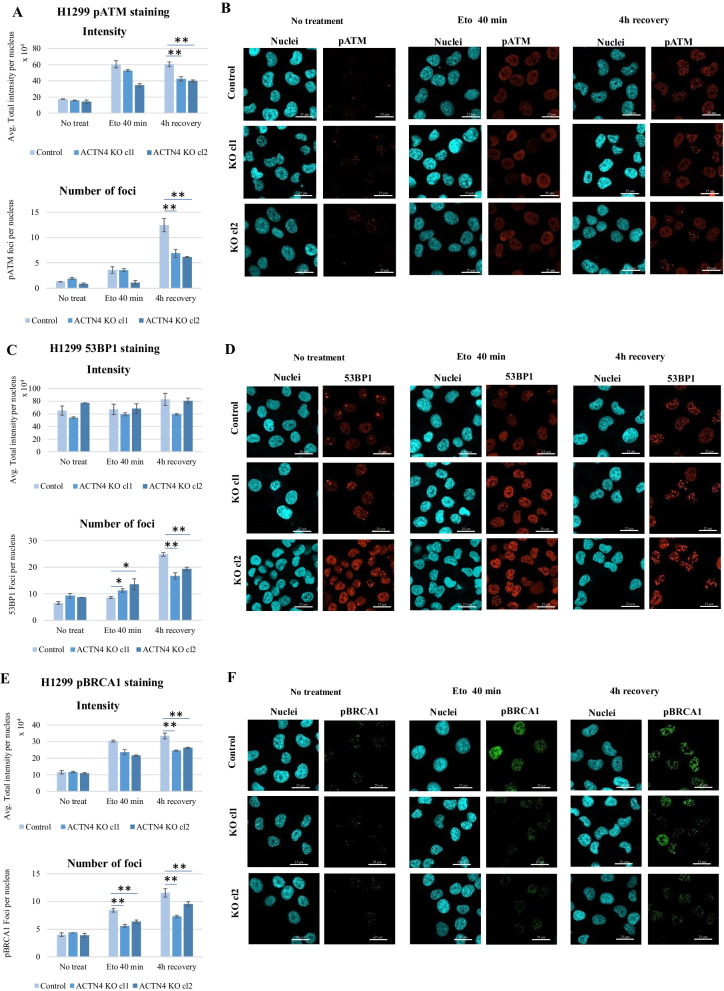
ACTN4 expression affects the distribution of the key DSBs repair proteins in H1299 cells after the etoposide-induced DNA mage. The H1299 Control and ACTN4 KO cells were treated with 50 uM etoposide for 40 min. The media was then replaced with a fresh one, and cells were incubated for 4 h to recover from the damage. Changes in total intensity and foci number of pATM (**A**–**B**), 53BP1 (**C**–**D**), pBRCA1 (**E**–**F**) proteins were estimated by immunofluorescence. Representative images are presented on **B**, **D** and **F**. Scale bars in panels represent 25 μm. Graphs **A**, **C**, **E** present the mean of three replicas ± SE. Over 500 cells were analyzed per replica. **p < 0.0001, **p < 0.001, *p < 0.05 compared to untreated cells (Student’s t-test)

To further elucidate ACTN4 involvement in the assembly of the major DSB repair complexes, we analyzed the distribution of phosphorylated ATM (pATM), 53BP1 and phosphorylated BRCA1 (pBRCA1) proteins during the DNA damage and repair [30]. ATM phosphorylation occurs early and participates in both NHEJ and HR pathways along with gamma-H2AX and 53BP1 [31]. By contrast, BRCA1 seemed to promote only one type of DNA repair, HR-mediated DSBs repair [32–35]. Hence, we expected that changes detected in the NHEJ/HR ratio may also lead to an altered distribution of the key repair proteins upstream of the DSB recognition itself. To test the hypothesis, we treated control H1299 cells and ACTN4 KO clones with 50 uM etoposide for 40 min, and let them realize the DNA repair program for 4 h. Re-distribution of pATM, 53BP1 and pBRCA1 proteins was analyzed by immunostaining. We found that number of pATM foci and total signal intensity in the nuclei did not correlate with the ACTN4 expression in untreated cells or after 40 min etoposide (Fig. 5A–B). Following 4 h of DNA repair, the ACTN4 KO clones demonstrated a significantly lower number of foci than control cells, similarly to what was found for gamma-H2AX staining (Fig. 3A). Thus, pATM distribution may reflect the enhanced DSB repair in the ACTN4 depleted cells with no differences in the DNA breaks recognition process.

Analysis of 53BP1 revealed statistically significant differences between the control and ACTN4 KO cells in the number of foci but not in the signal intensity (Fig. 5C–D). While the mean signal intensity per nucleus did not change in response to etoposide, the mean number of foci progressively elevated. However, the ACTN4 KO clones showed a higher foci number after 40 min incubation with the etoposide. These results coincide with enhanced NHEJ detected with the reporter plasmids. On the contrary, the number of foci was again higher in the control H1299 cells after 4 h recovery, which might result from the less efficient DSB repair.

**Fig. 6:**
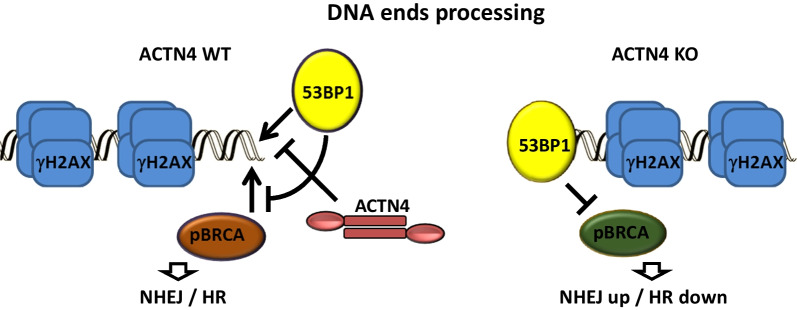
Possible mechanism of ACTN4 influence on the DSBs repair pathways. ACTN4 may possibly interfere with 53BP1 binding to unprocessed DNA ends, hence suppressing the end processing suitable for NHEJ and promoting assembly of the pBRCA-positive HR complexes. In H1299 ACTN4 KO cells however, DNA binding of 53BP1 is increased, which results in enhancing the NHEJ pathway and the suppression of pBRCA complexes and HR pathways

The pBRCA1 foci numbers were found to be approximately even in the H1299 control and ACTN4 KO cells with no treatment. Following the genotoxic stress, the ACTN4 knock-out cells showed a lower foci number both right after the etoposide treatment and after 4 h of repair (Fig. 5E–F). Thus, our data supports our hypothesis that the down-regulation of the ACTN4 gene in H1299 cells results in enhanced DSB repair, increasing the NHEJ/HR ratio. The differences in re-distribution of the early (gamma-H2AX, pATM) and late (53BP1, pBRCA1) acting proteins may suggest that ACTN4 is not involved in the break-points recognition but rather in a yet unknown process that regulates the balance between two major DSB repair systems in NSCLC cells (Fig. 6).

## Discussion

Our investigation revealed new aspects of ACTN4 functions in human cells, since the involvement of ACTN4 in the DSB repair process and, consequently, modulation of cancer cell resistance to genotoxic drugs have not yet been described. Importantly, our data coincides with clinical studies that identified ACTN4 as a predictive marker for the efficacy of the platinum-based therapy of NSCLC [24, 25]. Particularly, these studies have shown that the administration of adjuvant chemotherapy with cisplatin correlates with an overall survival in patients with high ACTN4 mRNA and protein levels. However, down-regulation of the ACTN4 gene expression does not increase the resistance of A549 lung cancer cells [24]. We have obtained similar results with the H1299 cell line. Future studies should reveal whether NSCLC cells with a high expression of ACTN4 can develop a sensitivity to cisplatin. Alternatively, ACTN4-mediated sensitivity to cisplatin may be governed by the immune system on the organismal level, given that ACTN4 is a transcriptional co-activator for NF-kappaB, a master regulator of immune cells [7].

Cisplatin causes DNA inter- and intra-strands cross-links in the cell, which then transform into the DSBs if not repaired before the cell enters the S-phase [36]. Thus, we have also tested cell resistance to topoisomerase II inhibitors, which induce DSBs directly [37]. According to the results obtained, both etoposide and doxorubicin are less effective DNA damage inducers in H1299 cells with the depletion of ACTN4 compared to wild-type cells. Moreover, the drug resistance seems to be the consequence of more effective DSB repair. Two independent methods, analysis of gamma-H2AX foci and the DNA comet assay, produce coherent results, which make the data more reliable. Specifically, ACTN4 knock-out cells displayed a reduced number of gamma-H2AX foci and a faster recovery of the DNA integrity after etoposide and doxorubicin treatment compared to control cells. Further, ACTN4 knock-out in another NSCLC cell line, H460, resulted in a more efficient recovery of DNA comets, which suggests that ACTN4 involvement in DSBs repair may be common for NSCLC cells, although it should be acknowledged that in H460 cells ACTN4 plays multiple roles. Importantly, the comet assay results show no effect of ACTN4 expression right after the drug treatment suggesting that DNA breaks are induced equally in the cells with and without ACTN4, suggesting that ACTN4 acts on the level of DNA damage response. No visible effect of ACTN4 depletion on the cell cycle before treatments also supports this assumption. Thus, we assume that low ACTN4 expression promotes a specific type of the DNA damage response, providing resistance to the topoisomerase II inhibitors but not to cisplatin.

Our hypothesis has been supported by the analysis of DNA repair pathways. Several types of repair may eliminate cisplatin-induced DNA damage, including nuclear and base excision repair (NER and BER, respectively), NHEJ and HR (see [38, 39] for review). The two later pathways are mainly used to correct the DNA strand breaks caused by the etoposide and doxorubicin treatment [40, 41]. Our data point out that ACTN4 is a novel player in the DNA damage response circuitry [42]. It acts as a regulatory factor affecting both of them in opposite ways, promoting HR and suppressing NHEJ. Mechanistically, participation of the ACTN4 protein in the DNA repair process still remains to be elucidated. One possibility is that it may influence the chromatin structure via interaction with the chromatin remodeling complex INO80 [3], which is known to be involved in transcriptional regulation, DNA replication, and DNA repair [43]. To test the hypothesis, we estimated the repair efficiency of the NHEJ and HR reporter plasmids following transient transfection and integration into the genome. Similar results were obtained in both cases, although integrated plasmids show much lesser efficiency overall, probably due to the restricted availability of DNA to the endonuclease cleavage. Thus, these data do not support a key role of the chromatin remodeling proteins in the ACTN4-dependent DSB repair.

Our preliminary analysis of the marker proteins involved in the recognition and processing of the DNA breaks reveals that ATM, 53BP1 and BRCA1 respond to genotoxic stress differently, depending on the ACTN4 expression. We do not observe a correlation between the ATM activation and clustering on the one hand and ACTN4 expression level after the etoposide treatment on the other hand. On the contrary, ACTN4 deficiency seems to facilitate the 53BP1 foci assembly, while activated BRCA1 (pBRCA1) shows the opposite trend. As the 53BP1 and BRCA1 proteins are considered to control NHEJ and HR, respectively [44], the results provide additional support to the hypothesis that ACTN4 mediated changes in the NHEJ/HR balance. Since H1299 cells, unlike H460 cells, lack p53, it would be interesting to see whether p53 contributes to the ACTN4-dependent regulation of NHEJ/HR. Further, if true, the next question is whether p53 activating small molecules can overcome the effect of ACTN4 [45–50]. However, our preliminary results do not support the idea of direct involvement of p53 in this regulation.

Our data also suggests that ACTN4 does not alter the primary recognition of DSBs but rather participates in the following enzymatic processing of the DNA ends, possibly interfering with the 53BP1 and BRCA1 binding to the DNA break-points. According to recent findings, 53BP1 appears to be a key regulator of DSB repair pathways, although the mechanisms of action remain to be largely elusive [44]. Our data may uncover a novel protein that controls the NHEJ/HR ratio just upstream of 53BP1 (Fig. 6).

Collectively, we demonstrated that a low expression of the ACTN4 gene facilitates the DSB repair in NSCLC cells, via enhancing NHEJ. Moreover, ACTN4 seems to control the choice between NHEJ and HR pathways, possibly regulating assembly of the 53BP1 and pBRCA1 complexes in response to DNA damage. Consequently, low-expressing NSCLC cells may be more resistant to DSB-inducing drugs. Further investigation of protein partners during the DNA damage response may uncover the ACTN4-mediated molecular events that control DSB repair in different subtypes of NSCLC [51].

## Conclusions

In summary, attenuation of ACTN4 expression in the NSCLC cell line, H1299, makes them more resistant to etoposide, the topoisomerase II inhibitor, but not to the platinum-based agent cisplatin. We hypothesize that ACTN4 is involved in DNA double-strand breaks repair in NSCLC cells, possibly by regulating the balance between the major DSBs repair pathways, NHEJ and HR. Our data also suggest that ACTN4 may interfere with the assembly of the 53BP1 and BRCA1 DNA repair complexes, thereby preventing the activation of NHEJ in favor of the less abundant HR pathway.

## Methods

### Cell cultures and plasmid manipulations

H1299, H460 and HEK293T cell lines were obtained from the American Type Culture Collection (ATCC). Cells were maintained in RPMI-1640 (H1299 and H460) or DMEM (HEK293T) media (Gibco, USA) supplemented with 10% heat-inactivated fetal bovine serum (Gibco, USA), 100 U/ml penicillin, 100 mg/ml streptomycin (Biolot, Russia), and 2 mM L-glutamine (Biolot, Russia). Cells were maintained at 37 °C in a humidified incubator with 5% CO_2_.

To perform the ACTN4 gene knockout in H1299 and H460 cells, we designed a sgRNA sequence using the CCTop CRISPR/Cas9 target online predictor tool (http://crispr.cos.uni-heidelberg.de). The sgRNA encoding oligonucleotides (5′-CACCTGGGGCCGTACTGGTACGAC-3′ and 5′-ACCCCGGCATGACCATGCTGCAAA-3′) were annealed and cloned into the lentiCRISPRv2 vector between sites for the BsmBI endonuclease. Lentiviral particles were produced by the transfection of HEK239T cells in 10-cm Petri dishes with the lentiCRISPRv2 ACTN4 knock-out construct (10 ug) along with the packaging plasmids psPAX2 (8 ug) and pMD2.G (4 ug). The virus-containing media were collected at 48–72 h post transfection. The filtered medium was then added to the target cells. Following 24 h of incubation, the medium was replaced with a fresh one, containing 1.5 ug/ml puromycin. The cells were selected on puromycin for 10 days, individual clones were obtained and tested for the ACTN4 protein level. To generate control cells, the lentiCRISPRv2 construct with no specific sgRNA sequence was used with no further sub-cloning of the transduced cells.

To generate ACTN4 overexpression, we amplified the coding sequence, using Phusion High-Fidelity DNA Polymerase (NEB) and primers 5′-GAGAATTCGGAATGGTGGACTACCAC-3′ and 5′-CTGCGGCCGCTCACAGGTCGCTCTCGC-3′. The amplified sequence was inserted into the pCDH-EF1 vector, using restriction endonucleases EcoRI and NotI. The plasmid was packed into lentiviral particles and used for transduction of ACTN4 coding sequence into H1299 cells as described above. Following selection on puromycin for 14 days, ACTN4 overexpression was tested by immunoblotting. The cells with an empty pCDH-EF1 vector were used as controls.

All transfections were performed with the Turbofect reagent (Thermo Scientific) according to the manufacturer’s protocol. Plasmids were purified by the GeneJET plasmid miniprep kit (Thermo Scientific).

### Cell cycle and apoptosis analysis

The cell cycle analysis was performed by flow cytometry. The approximate number of cells was 0.5–1 × 10^6^ per sample. Harvested cells were washed with PBS, resuspended in 0.5 ml of PBS, and fixed with 0.5 ml of 96% ice-cold ethanol added dropwise with vortexing to avoid cells clumping together. The samples were then incubated for 20 min at − 20 °C, pelleted by centrifugation at 2000 rpm for 5 min, and the tubes were inverted to decant the ethanol traces. The fixed cells were incubated in PBS with 40 ug/ml Propidium Iodide (PI) and 100 ug/ml RNAse A (Thermo Scientific) at 37 °C for 30 min.

The apoptosis rate was analyzed using Annexin V conjugated with Alexa Fluor 647 (A23204, Thermo Fisher Scientific) and PI. The cells were treated with 50 uM etoposide for 24 h. Cells were harvested, washed with PBS, and resuspended at 1 × 10^6^ cells/ml in the annexin-binding buffer (10 mM HEPES, pH 7.4, 0.14 M NaCl, 2.5 mM CaCl2). 100 ul of the cell suspension were incubated with Annexin V (5 ul) and PI (1 ug/ml) for 15 min. The cells were then diluted with an annexin-binding buffer (400 ul).

Flow cytometry was performed using the CytoFlex Flow Cytometer (Beckman Coulter), and the cell cycle was analyzed using CytExpert v2.3.1.22 software.

### Cytotoxicity assay

Cell viability was measured by an MTT assay. The cells were seeded at a density of 3 × 10^3^ per well in 96 well plates one day before their treatment with the genotoxic agents. The treatments were performed for 72 h. The 3-(4,5-dimethylthiazol-2-yl)-2,5-diphenyltetrazolium bromide (MTT) solution was then added to each well in a final concentration of 0.5 mg/ml. Subsequently, the wells were incubated for 4 h in the dark at 37 °C. The medium was removed, formazan was dissolved in DMSO, and the optical density was measured at 570 nm using the iMark microplate absorbance reader. For reference a wavelength of 630 nm was used.

### Comet assay

To induce genotoxic stress, the cells (15 × 10^4^ per 35 mm dish) were treated with 50 µM etoposide or 1.5 uM doxorubicin for 40 min. The medium was then replaced for a fresh one, and the cells were left in the CO2 incubator to repair the DNA breaks. Following the 8 h of incubation, the cells were trypsinased and washed with PBS. A sample of 10^4^ cells, thoroughly resuspended in 10 µl PBS, was mixed with 0.5% low melting agarose at 37 °C. One day before the experiment, the microscope slides (Menzel-Gläser, GmbH) were immersed into a hot 1% agarose solution and dried overnight at room temperature. The samples were placed onto the agarose pre-coated slides, and dried for 10 min at + 4 °C. The slides were then incubated in the lysis buffer (2.5 M NaCl, 0.1 M EDTA, 10 mM Tris–HCl, pH 10.0, 1% Triton X-100) for 1 h at + 4 °C followed by incubation with the alkaline unwinding buffer (0.2 M NaOH, 1 mM EDTA pH 10.0) for 20 min in the dark at room temperature. Then, slides were rinsed with ice-cold water, subjected to electrophoresis at 23 V for 30 min in a standard 23 cm chamber in an ice-cold buffer (0.2 M NaOH, 1 mM EDTA), and stained with 1:10,000 SYBR Green II (Sigma-Aldrich). Images were analyzed by Open Comet software. More than two hundred cells per sample were evaluated. Experiments were repeated at least three times showing the same trend.

### Immunofluorescence

Immunostaining for DNA repair proteins was performed as described previously [52]. Briefly, 4 × 10^3^ cells per well were inoculated in 96-well plates (Eppendorf, GmbH). The next day, the cells were treated with 50 µM etoposide or 1.5 uM doxorubicin for 40 min, followed by incubation with a fresh medium for 4 h to allow the cells to repair the DNA damage. The cells were washed with cold PBS and fixed in 4% paraformaldehyde for 30 min. After washing with PBS, the cells were permeabilized with 0.5% Triton X-100 in PBS for 15 min, treated with the blocking buffer (0,5% Triton X-100, 2% BSA in PBS) for 30 min, and incubated with primary antibodies for 1.5 h. The antibodies against gamma-H2AX (9718, Cell Signaling, USA), 53BP1 (NB100-304, Novus Biologicals, USA), pBRCA1 (9009, Cell Signaling, USA), pATM (Ser1981) (ab36810, Abcam, UK) were used. After being washed 3 times with the blocking buffer, the samples were incubated with the Alexa 488- or 546-conjugated secondary antibodies for 40 min. Nuclei were stained with Hoechst 33,342 (4 ug/ml). Image capture and signal quantifications were performed with the CellVoyager CQ1 High-Content Analysis System (Yokogawa, Japan).

### Western blotting

Immunoblotting was performed as described elsewhere [16]. The cells were harvested and washed with PBS, then lysed on ice with RIPA lysis buffer (10 mM Tris–HCl, pH 8.0, 1 mM EDTA, 0.5% Triton X-100, 0.5% SDS, 140 mM NaCl) containing 1 mM PMSF, a cocktail of protease inhibitors (Roche) for 10 min. The lysates were then sonicated and centrifuged at 14,000 g for 15 min. The protein concentration was determined with a Pierce™ BCA Protein Assay Kit (Thermo Scientific) according to manufacturer’s protocol. Protein samples of 10 mg were separated by Laemmli SDS-PAGE, and transferred to a PVDF membrane (Millipore). The membrane was blocked with 5% nonfat milk, probed with specific primary antibodies, and then with HRP-conjugated secondary anti-mouse or anti-rabbit IgG antibodies (Sigma-Aldrich, USA). The signals were visualized with luminol and paracumarin using the ChemiDoc detection system (BioRad, USA). The antibodies against ACTN4 (0042-05, ImmunoGlobe GmbH) and beta-actin AC-15 (ab6276, Abcam) were applied.

### Reporter plasmids for the DSB repair pathways analysis

All manipulations were carried out as described previously [53, 54]. Briefly, 15 × 10^3^ cells per well were inoculated into a 48-well plate. The cells were co-transfected with 0.15 ug NHEJ- or 0.6 ug HR-reporter plasmids, and plasmid encoding I-SceI endonuclease was fused with dsRed protein (0.1 ug or 0.4 ug, correspondingly). The ISceI-GR-RFP plasmid was a gift from Tom Misteli (RRID: Addgene 17654). The pcDNA3.0 plasmid was used as a negative control for excluding auto-fluorescent cells. Simultaneously with transfection, 100 nM triamcinolone acetonide (Sigma-Aldrich) was added into the medium to induce the nuclear translocation of the endonuclease [55]. Efficiency of the GFP gene repair in the reporter plasmids was evaluated by the CytoFlex Flow Cytometer 3 days after transfection using a green-versus-red fluorescent plot. The number of dsRed-positive cells was used to assess the transfection efficacy. Typically, 10^4^ cells were analyzed in each sample. DNA repair efficiency was estimated as the ratio of GFP + /Red + cells. Also the assay was performed on the H1299 cells encoding the NHEJ or HR reporter cassette in the genome according the procedures described [29].

### Statistical analysis

All presented images of immunofluorescence and cell-cycle assay are representative of at least three independent experiments. Relative quantification cell viability assays were performed in triplicate, and each experiment was repeated three times. The data shown are the mean ± S.E. of at least three replicates from one experiment. Statistical significance was considered at *p* < 0.05 using Student’s t-test.

## Supplementary Information

**Additional** **file** **1. Figure S1**: ACTN4 expression determines the DSBs repair pathway in the chromatin context. H1299 control and ACTN4 KO cl1 cells with integrated NHEJ and HR specific reporter constructs were transfected with the dsRed-fused I-SceI encoding plasmid. Representative FACS plots are presented. The graph represents DNA repair efficiency estimated as the ratio of GFP+/dsRed+ cells. Data are presented as mean of three replicas ± SD. ***p < 0.0001compared to untreated cells (Student’s t-test).

## Data Availability

Data sharing is not applicable to this article as no datasets were generated or analysed during the current study.

## Abbreviations

53BP1: 53-Binding protein 1
ACTN4: Alpha-actinin-4
ATM: Ataxia telangiectasia mutated
BRCA1: Breast cancer gene 1
DSB: Double strand break
FSGS: Focal segmental glomerulosclerosis
GFP: Green fluorescent protein
GR: Glucocorticoid receptor
HDAC7: Histone deacetylase 7
hnRNP: Heterogeneous nuclear ribonucleoprotein
HR: Homology recombination
KRAS: Kirsten rat sarcoma virus
MTT: 3-(4,5-Dimethylthiazol-2-yl)-2,5-diphenyltetrazolium bromide
NHEJ: Non-homologous end joining
NF-kappaB: Nuclear factor kappa-light-chain-enhancer of activated B cells
NSCLC: Non-small cell lung cancer
RFP: Red fluorescent protein
