# Supplementary figures and images for: Alpha-actnin-4 (ACTN4) selectively affects the DNA double-strand breaks repair in non-small lung carcinoma cells
Source: Biol Direct. 2022 Dec 7;17:40. doi: 10.1186/s13062-022-00354-6 (PMC9730676; doi:10.1186/s13062-022-00354-6)

Suppl. fig 1

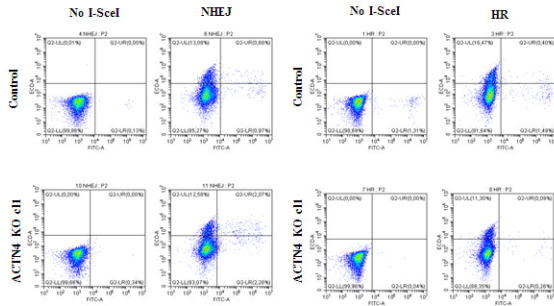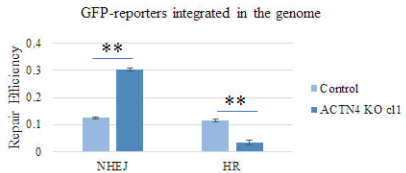

Supplement: Supplementary file 1 — Additional file 1. Figure S1: ACTN4 expression determines the DSBs repair pathway in the chromatin context. H1299 control and ACTN4 KO cl1 cells with integrated NHEJ and HR specific reporter constructs were transfected with the dsRed-fused I-SceI encoding plasmid. Representative FACS plots are presented. The graph represents DNA repair efficiency estimated as the ratio of GFP+/dsRed+ cells. Data are presented as mean of three replicas ± SD. ***p < 0.0001compared to untreated cells (Student’s t-test). [file 13062_2022_354_MOESM1_ESM.pdf]
